# Purification, Structural Characterization, and Immunomodulatory Activities of a Glucan from *Morchella sextelata*

**DOI:** 10.3390/foods15010105

**Published:** 2025-12-29

**Authors:** Shiqiong Xiang, Yang Chen, Jiayue Xia, Guiju Sun

**Affiliations:** 1 Key Laboratory of Environmental Medicine and Engineering of Ministry of Education, Department of Nutrition and Food Hygiene, School of Public Health, Southeast University, Nanjing 210009, China; xiangshiqiong@163.com (S.X.);; 2 School of Biology and Food Engineering, Chongqing Three Gorges University, Chongqing 404000, China

**Keywords:** *Morchella sextelata*, glucans, structural characterization, immunomodulatory activity

## Abstract

In search of natural and safe compounds with immunomodulatory effects, this study identified a glucan with a molecular weight (Mw) of 1.2 × 10^7^ Da, named MSP-1-1, which was extracted and purified from *Morchella sextelata* via water extraction, alcohol precipitation, and column chromatography. Based on comprehensive characterization using HPAEC, SEC-MALLS-RI, FT-IR, GC-MS, NMR, and SEM, a structural model for MSP-1-1 is proposed. The model depicts a glucan with a backbone predominantly composed of →4)-α-D-Glc*p*-(1→ linkages, featuring occasional →4,6)-α-D-Glc*p*-(1→ residues that serve as branch points. The branches are identified as single α-D-Glc*p*-(1→ units attached at the O-6 position of these branching residues. In vivo experiments revealed that MSP-1-1 restored cyclophosphamide-induced abnormalities in immune organ indices, histology, and peripheral blood parameters. Additionally, MSP-1-1 significantly enhanced macrophage phagocytosis, splenic lymphocyte proliferation, and the proportions of CD3^+^CD4^+^ and CD3^+^CD8^+^T cells, while increasing the CD3^+^CD4^+^/CD3^+^CD8^+^ ratio. It also elevated concentrations of IgA and IgM in both serum and thymus, indicating immunomodulatory activity. In summary, this research elucidated the structural characteristics and immunomodulatory activity of MSP-1-1, providing insights into the bioactivity of *M. sextelata* glucan and a basis for further exploring its potential functional applications.

## 1. Introduction

As an edible fungus, the morel mushroom (*Morchella* spp., Pezizales, Ascomycota) has been used for centuries as a common food, medicine, and flavoring agent because of its delicious flavor and nutrients [1]. As the main active ingredient of *Morchella*, glucans have various bioactive functions—for instance, antitumor and immunomodulatory activities [2,3,4]. However, wild morels are affected by environmental factors, leading to high prices and low yields. The varieties of *Morchella* successfully cultivated in China include *Morchella importuna*, *Morchella sextelata,* and *Morchella septimelata* [5]. Due to the short period of successful artificial cultivation of *M. sextelata*, there has been limited research focusing on isolating glucans from artificially cultivated *M. sextelata* and studying their structures and bioactivities [6,7].

Glucans, as members of the polysaccharide family, are commonly found in edible mushrooms and exhibit biological activities, including antioxidant, immunomodulation, and anti-tumor. Among these, immunomodulation is one of the primary biological activities. Studies have exhibited that glucans from various sources with immunomodulatory activity share similar glycosidic bonds [8,9,10]. Polysaccharides can interact with the immune system through multiple pathways, triggering intercellular communication and molecular recognition, thereby inducing an immunostimulatory response. This is demonstrated by the ability of polysaccharides to directly regulate immune responses via multiple levels and pathways, or indirectly modulate the organism’s immune system through interactions with the gut microbiota [11,12].

Research has demonstrated that the conformation and type of glycosidic bond in polysaccharides can affect their immunomodulatory activity [13]. Depending on the glycosidic bond, mushroom polysaccharides can be separated into α-glucans, β-glucans, and heteroglycans [14]. The triple-helix structure is more common in β-glucans and may possess higher immunomodulatory activity [15]. However, recent studies have shown that an α-glucan extracted from Hirsutella sinensis mycelium dramatically enhances phagocytosis by macrophages via the activation of p38, JNK, and NF-κB signaling pathways. This is attributed to the unique structure of the α-1,4 glycosidic linkages within the polysaccharide, which contain branched glucose residues in the O-6 position, terminating in glucose [16]. In summary, edible mushroom glucans exhibit diverse types and complex structures, with significant differences in their biological activities. Currently, the structure of glucans from *M. sextelata* remains unclear, and their immune-regulatory activity has not been thoroughly investigated, which limits further development and utilization of *M. sextelata* glucans.

The edible mushroom *M. sextelata* is valued for its nutritional and potential bioactive properties; however, the structural characteristics of its glucan fractions and their immunomodulatory activities remain poorly elucidated. Notably, the structure–function relationship of *M. sextelata*-derived glucans—a critical basis for their development as functional food ingredients—has not been systematically explored. To address these research gaps, this study intended to clarify the chemical structure of a purified glucan fraction from *M. sextelata* and evaluate its immunomodulatory potential. Specifically, we extracted glucans from *M. sextelata* and purified them to a homogeneous fraction via sequential gel-filtration and ion-exchange chromatography. Their detailed chemical structure was analyzed, and their immunomodulatory impacts were assessed using a cyclophosphamide (CTX)-induced immunosuppressed mouse model. This work aims to bridge the existing knowledge gap in understanding the structure–function relationship of *M. sextelata* glucans while laying a theoretical framework for the rational exploitation and utilization of *M. sextelata* as an immunity-boosting functional food resource.

## 2. Materials and Methods

### 2.1. Materials

*M. sextelata* powder was obtained from Nanhua County, Yunnan Province, China. DEAE (diethylaminoethyl) SepLife FF was purchased from Xi’an Lanxiao Technology New Materials Co., Ltd. (Xi’an, China). Sephacryl S-400HR was provided by GE Healthcare (Boston, MA, USA). Thirteen monosaccharide standards, including galactose (Gal), fucose (Fuc), rhamnose (Rha), glucose (Glc), xylose (Xyl), arabinose (Ara), fructose (Fru), ribose (Rib), mannose (Man), glucuronic acid (GlcA), galacturonic acid (GalA), guluronic acid (GulA), and mannurionic acid (ManA) (purity ≥ 99.9%), were purchased from Sigma-Aldrich (St. Louis, MO, USA). Additionally, lipopolysaccharide (LPS) from E. coli serotype O55:B5 (purity > 97%, Cat. No. L2880) and Concanavalin A (ConA) lyophilized powder (Type IV, Cat. No. C2010) were also obtained from the same supplier. Cyclophosphamide (Cat. No. C126044, purity > 98%) and levamisole hydrochloride were derived from Aladdin Biochemical Technology Co., Ltd. (Shanghai, China). Beyotime Biotechnology Co., Ltd. (Shanghai, China) provided Ammonium Chloride-Potassium Chloride (ACK) lysis buffer and CCK-8 (Cat. No. C0037). ELISA kits for immunoglobulins, namely, immunoglobulin M (IgM, Cat. No. LA128811H) and immunoglobulin A (IgA, LA128859H), were supplied by Nanjing LaPuDa Biotechnology Co., Ltd. (Nanjing, China). Fetal bovine serum (FBS) and RPMI-1640 medium were supplied by Solarbio Science &Technology Co., Ltd. (Beijing, China), along with PE anti-mouse CD3 Antibody, AB_312662 (BioLegend Cat. No. 100205); FITC anti-mouse CD4 Antibody, AB_312690 (BioLegend Cat. No. 100405); and PE/Cyanine5 anti-mouse CD8a Antibody, AB_312749 (BioLegend Cat. No. 100710).

### 2.2. Extraction and Purification of MSP

**Figure 1:**
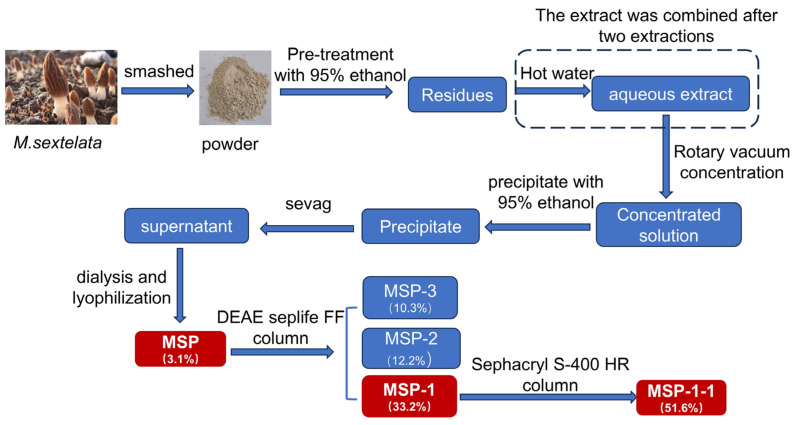
Preparation process of *M. sextelata* glucans.

The extraction protocol for MSP was adapted from a previous method [17], as illustrated in Figure 1. Briefly, powdered *M. sextelata* obtained through cell wall disruption was mixed with 95% (*v*/*v*) at a 1:20 *w*/*v* ratio, then refluxed at 60 °C for 120 min to remove pigments and fats. After removing the supernatant, the remaining solid was combined with 20 volumes of distilled water and refluxed for two hours at 80 °C. The process was performed twice. After extraction, a rotary evaporator (set to 60 °C) was applied to concentrate the supernatant to one-fifth of its initial volume. The concentrate was then held at 4 °C for 12 h prior to centrifugation, and a four-fold amount of 95% (*v*/*v*) ethanol was incorporated. The crude polysaccharide (MSP) was obtained by dialyzing the precipitates for 72 h (molecular weight cut-off: 3500 Da), deproteinizing them with Sevag reagent (chloroform/n-butanol, 4:1, *v*/*v*), and finally lyophilizing the product.

The three fractions, namely, MSP-1, MSP-2, and MSP-3, were acquired by loading MSP onto a DEAE SepLife FF column (26 mm × 400 mm) and performing stepwise elution with a gradient system consisting of deionized (DI) water and NaCl solutions of 0.1, 0.2, and 0.3 mol/L at a flow rate of 4 mL/min. These fractions were subsequently subjected to dialysis and lyophilization. The highest-yielding fraction (MSP-1) was chosen for further purification utilizing a 26 mm × 1000 mm Sephacryl S-400HR column with DI water as the eluent at 1.0 mL/min, yielding a homogeneous glucan fraction (MSP-1-1).

### 2.3. Structural Characterization of MSP-1-1

#### 2.3.1. FT-IR and UV-Vis

With the use of the KBr pellet method, FT-IR spectra were acquired, in which MSP-1-1 was milled and mixed with spectroscopic-grade KBr. The scanning measurements were conducted with an IR spectrometer (Nicolet iS10, Thermo Fisher Scientific, Waltham, MA, USA) between 4000 and 400 cm^−1^ [18].

The UV-Vis spectrum of MSP-1-1 aqueous solution (5 mg/mL) was measured with a multifunctional microplate reader (Multiskan GO, Thermo Fisher Scientific, Waltham, MA, USA). Scanning was conducted between 200 nm and 1000 nm at 1 nm intervals. DI water was employed as the blank control, and reference measurements were carried out under identical parameters [19].

#### 2.3.2. Molecular Weight (Mw) Analysis

MSP-1-1 was solubilized in 0.1 M NaNO3 (with 0.02% NaN_3_, *w*/*w*) to yield the ultimate concentration (1 mg/mL). The Mw and homogeneity of the fraction were determined using SEC-MALLS-RI; this analytical system comprised a UHPLC system (UltiMate 3000, Thermo Fisher Scientific, Waltham, MA, USA), paired with an Optilab T-rEX refractive index detector (Wyatt Technology, Santa Barbara, CA, USA) and a DAWN HELEOS II multi-angle light scattering detector (Wyatt Technology, Santa Barbara, CA, USA). Two 300 × 8 mm gel permeation chromatography columns (SB-803 HQ and Shodex OHpak SB-805 HQ) were connected in sequence. The mobile phase was composed of 0.02% NaN_3_ and 0.1 M NaNO_3_; the injection volume was set to 100 μL, the flow rate was set to 0.6 mL/min, and the column temperature was maintained at 45 °C [20].

#### 2.3.3. Monosaccharide Composition Analysis

First, an appropriate amount of MSP-1-1 and monosaccharide standards was meticulously weighed and transferred into a chromatography vial. After adding 1 mL of a 2 M TFA solution, the mixture was incubated for two hours at 121 °C. Post-hydrolysis, the samples were evaporated to dryness under a nitrogen stream, then rinsed with 99.99% methanol; this rinsing process was repeated 2–3 times, with thorough drying between each step. The sample was then moved to a sterile chromatography vial for analysis after being dissolved in sterile water.

The Thermo Scientific ICS 5000+ ion chromatography system (Thermo Fisher Scientific, Waltham, MA, USA) with a Dionex CarboPac PA20 column (150 × 3.0 mm, 10 μm) was exploited to analyze the MSP-1-1 monosaccharide composition [21]. Separation was conducted at 30 °C with a flow rate of 0.5 mL/min. Three mobile phases were used: (A) ultrapure water, (B) 0.1 M NaOH, and (C) 0.1 M NaOH supplemented with 0.2 M sodium acetate. The gradient elution program was configured as follows: 0 min, A/B/C (95:5:0, *v*/*v*); 26 min, A/B/C (85:5:10, *v*/*v*); 42 min, A/B/C (85:5:10, *v*/*v*); 42.1 min, A/B/C (60:0:40, *v*/*v*); 52 min, A/B/C (60:40:0, *v*/*v*); 52.1 min, A/B/C (95:5:0, *v*/*v*); 60 min, A/B/C (95:5:0, *v*/*v*).

#### 2.3.4. Methylation Analysis

First, MSP-1-1 (2–3 mg) was solubilized in 500 μL of DMSO, then NaOH (1 mg) was incorporated, followed by incubation for 30 min. Then, 50 μL of methyl iodide was introduced to achieve full methylation, and the reaction proceeded for 1 h. After adding dichloromethane (2 mL) and water (1 mL) and mixing, the organic phase was isolated via centrifugation and dried under a nitrogen gas stream. Then, 2 M TFA (100 μL) was added for hydrolysis at 121 °C, and the sample was concentrated post-reaction. Subsequently, 50 μL of 1 M NaBD_4_, along with 2 M ammonia solution, was combined with the sample, and the reaction ran for 2.5 h. The reaction was stopped by adding 20 μL of acetic acid, and the sample was dried under nitrogen. This was followed by two washes with 250 μL of 1:3 ethanol each, with the sample dried under nitrogen between each wash step. After adding 250 μL of acetic anhydride, the mixture was acetylated at 100 °C for 2.5 h. A total of 1 mL of water was added, and the mixture was allowed to sit for 10 min. Dichloromethane was applied to extract the organic phase, the aqueous phase was disposed of, and the organic phase was analyzed with GC-MS [22,23].

GC-MS analysis was carried out using an Agilent 6890A-5977B system (Agilent Technologies, Santa Clara, CA, USA) with an automatic sampler (model G4567A). Separation was carried out using a BPX70 capillary column (30 m × 0.25 mm × 0.25 µm film thickness; SGE, Ringwood, Victoria, Australia). High-purity helium served as the carrier gas. The injection volume was 1 µL with a split ratio of 10:1. The column oven temperature was first maintained at 140 °C for 20 min, then ramped up to 230 °C at 3 °C per minute, and lastly held at 230 °C for 3 min.

#### 2.3.5. NMR Spectroscopy

MSP-1-1 was dissolved in 0.5 mL of deuterium oxide (D_2_O) at a concentration of 40 mg/mL, followed by repeated D_2_O exchange and lyophilization cycles. Subsequently, at 25 °C, the ^1^H NMR, ^13^C NMR, correlation spectroscopy (COSY), heteronuclear single quantum coherence (HSQC), heteronuclear multiple bond correlation (HMBC), and nuclear Overhauser effect spectroscopy (NOESY) spectra were acquired using a Bruker AVANCE NEO 500 MHz spectrometer (Bruker, Rheinstetten, Germany) [24].

#### 2.3.6. SEM Observation

After passing through a 100-mesh screen, a small quantity of the MSP-1-1 samples was put onto conductive carbon tape. A SEM (Zeiss Merlin Compact, Oberkochen, Germany) in STEM mode was exploited to examine the morphology of MSP-1-1 following gold sputter coating [25].

### 2.4. Immunological Regulation

#### 2.4.1. Animals and Experimental Design

Specific pathogen-free (SPF) male BALB/c mice (6 weeks old, 20 ± 2 g, n = 90) were provided by Jinan Pengyue Experimental Animal Breeding Co., Ltd. (Jinan, China; certificate number: SCXK 2022-0006). All of the animal welfare and experimental procedures were in line with relevant ethical guidelines and authorized by the Animal Ethics Committee of Southeast University (approval No. 20230818001). Mice were caged in the SPF animal house at the School of Public Health of Southeast University, with 4–6 mice per cage. The rearing conditions were as follows: temperature 23–26 °C, relative humidity 40–70%, and a 12 h light:12 h dark photoperiod. Throughout the housing period, the mice were given ad libitum access to food and water.

**Figure 2:**
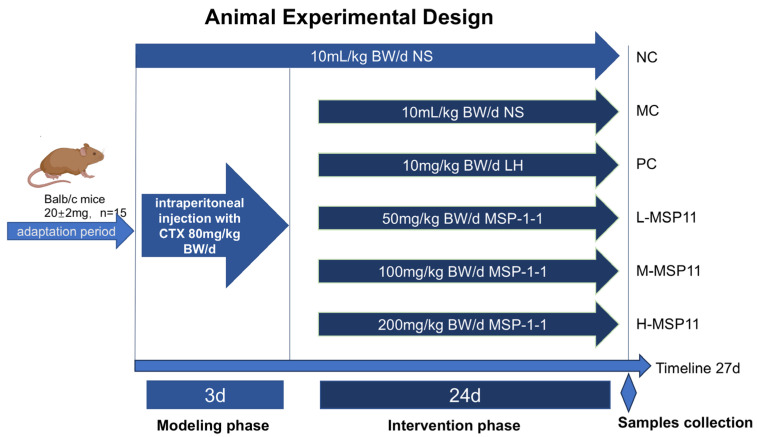
Schematic diagram of animal experimental design.

Following acclimatization, mice were randomized into 6 groups (n = 15/group; Figure 2). Five groups of mice, positive control (PC), model control (MC), high-dose MSP-1-1 (H-MSP11), medium-dose MSP-1-1 (M-MSP11), low-dose MSP-1-1 (L-MSP11), and were administered intraperitoneally with CTX at 80 mg/kg bw/day for 3 days in order to develop immunodeficient mouse models; the remaining groups of mice (negative control group, NC) were injected intraperitoneally with 10 mL/kg of saline as control. After successful model establishment, a 24-day oral gavage intervention was administered as follows: NC and MC, 10 mL/kg of saline; PC, 10 mg/kg bw/d levamisole hydrochloride (LH); L-MSP11, 50 mg/kg bw/d MSP-1-1; M-MSP11, 100 mg/kg bw/d MSP-1-1; and H-MSP11, 200 mg/kg bw/d MSP-1-1. During the intervention period, to prevent the recovery of immune function, in the NC group, mice received injections of 10 mL/kg of saline every 10 days, whereas all other groups—aside from the NC group—had injections of CTX (60 mg/kg bw/d). Mice were weighed every two days, and daily food intake was measured during the experiment to track body weight variations and dietary consumption across groups. All mice were fasted for 12 h (with unrestricted access to water), anesthetized via isoflurane inhalation, followed by blood collection via enucleation and dissection.

#### 2.4.2. Immune Organ Index

After dissecting the mice, the thymus and spleen were weighed individually. The organ indices were then calculated using the following formulas:

Thymus index (mg/g) = Thymus weight (mg)/Body weight of the mouse (g)

Spleen index (mg/g) = Spleen weight (mg)/Body weight of the mouse (g)

#### 2.4.3. Hematoxylin and Eosin (HE) Staining of the Spleen

The spleen tissues of mice were preserved by immersing them in 10% neutral buffered formalin, then dehydrated via a succession of ethanol grades before being embedded in paraffin. Sections of tissue were stained using H&E and investigated morphologically under a light microscope.

#### 2.4.4. Carbon Clearance Capacity Test

Mice were subjected to 12 h of fasting prior to tail vein injection with India ink (fourfold diluted in saline) at a dosage of 10 mL/kg bw. A total of 20 μL of blood was extracted at 2 and 10 min. At a wavelength of 600 nm, the OD values of the blood samples were determined after being diluted with 0.1%Na_2_CO_3_ solution (2 mL), which was utilized as a zeroing blank. The formulae were applied to detect the clearance rate (K) and phagocytic index (α).

$$\mathrm{clearance}\mathrm{rate}\;(K)=(\log{OD}_{1}-\log{OD}_{2})/(t_{2}-t_{1})$$

$$\mathrm{phagocytic}\;\mathrm{index}\left(\alpha \right)=\mathrm{body}\;\mathrm{weight}/(\mathrm{liver}\;\mathrm{weight}+\mathrm{splenic}\;\mathrm{weight})\times K^{1/3}$$

Note: OD_1_ and OD_2_ denote the absorbance at 2 min and 10 min, respectively; t_1_ and t_2_ denote the time points at 2 min and 10 min, respectively.

#### 2.4.5. Blood Component Analysis

Complete blood count was analyzed using a hematology analyzer (BC-5000Vet, Mindray, Shenzhen, China). The indices included white blood cell (WBC), red blood cell (RBC), hemoglobin (HGB), lymphocyte (Lym), neutrophil (Neu), and platelet (PLT) counts.

#### 2.4.6. Spleen Lymphocyte Proliferation Assay

In order to produce a single-cell solution, the spleen was aseptically separated and passed through a 200-mesh cell sieve. RBC lysis buffer was incorporated after the supernatant was removed following five minutes of centrifugation at 300× *g*. After incubating for 15 min, the lysis was terminated with phosphate-buffered saline (PBS). This process was repeated until complete lysis of erythrocytes was achieved. After that, the cells underwent three PBS washes. The cell density was adjusted to 2 × 10^6^ cells/mL after the cells were resuspended in RPMI-1640 media with 1% penicillin–streptomycin and 10% FBS. After seeding cell suspension (100 µL) into each well in a 96-well plate, 100 µL of either LPS solution (10 µg/mL) or ConA solution (5 µg/mL) was added. There were control wells with no mitogens. The plate was cultivated in an environment containing 5% CO_2_ for 48 h at 37 °C. Following incubation, each well received 20 µL of CCK-8 solution, and the plate was inoculated for a further 4 h under the same circumstances. A microplate reader was applied to detect the OD at 450 nm. Proliferation of lymphocytes was determined by subtracting the OD value of control wells (without mitogens) from that of stimulated wells (with ConA or LPS).

#### 2.4.7. Determination of Splenic T Lymphocyte Subpopulations via Flow Cytometry

The density of splenic lymphocytes was set at 2 × 10^6^ cells/mL. The supernatant was then discarded by centrifuging 200 µL of the cell solution. After rinsing twice with PBS, 0.5 μL of anti-CD4 antibody, 1.25 μL of anti-CD3 antibody, and 0.5 μL of anti-CD8a antibody were added to each tube. The mixture was gently mixed and incubated at 4 °C in the dark for 30 min. The reaction was stopped by adding PBS, and the cells were washed twice more to eliminate unbound antibodies. Finally, the cells were resuspended in 0.4 mL of PBS. An Attune NXT flow cytometer (Thermo Fisher Scientific, Waltham, MA, USA) was used to measure the proportions of CD3^+^, CD4^+^, and CD8^+^ T lymphocyte subsets.

#### 2.4.8. Measurement of Immunoglobulin Level in Serum and Thymus

The ocular sinuses of mice were utilized to draw blood, which was then centrifuged at 4 °C to extract serum for later use. A total of 20 mg of thymus tissue was collected and homogenized with pre-cooled PBS at a 1:9 (*w*/*v*) ratio, followed by centrifugation to collect the tissue homogenate supernatant. Thymus and serum levels of IgM and IgA were evaluated with commercially available ELISA kits, and immunoglobulin levels were computed based on standard curves created from the standards supplied with the kits. All procedures were implemented strictly as per the protocols of the manufacturer.

#### 2.4.9. Statistical Analysis

Statistical analyses were conducted using SPSS 24.0 (IBM Corporation, Armonk, NY, USA). The normality of the experimental data was evaluated via the Shapiro–Wilk test. Continuous variables that followed a normal distribution were presented as mean ± standard deviation. After verification of homogeneity of variance using Levene’s test, a one-way ANOVA was applied, followed by the LSD post hoc test. A *p*-value < 0.05 was regarded as statistically significant.

## 3. Results

### 3.1. Extraction, Purification, and Structure Characterization of MSP-1-1

#### 3.1.1. Extraction and Purification of MSP-1-1

**Figure 3:**
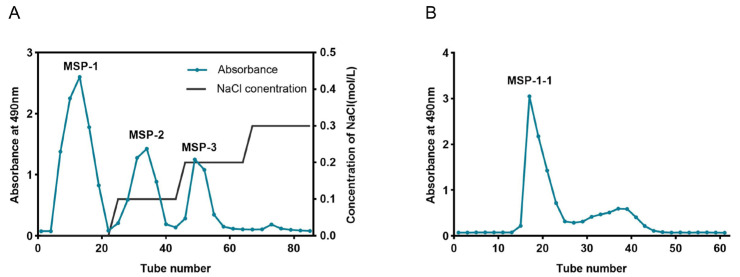
(**A**) MSP eluate on a DEAE SepLife FF column; (**B**) MSP-1 eluate on a Sephacryl S-400HR column.

The MSP isolated from *M. sextelata* achieved a yield of 3.1% (calculated relative to the dry powder weight of *M. sextelata*). Then, MSP was fractionated through anion exchange chromatography using a DEAE SepLife FF column; under our experimental conditions, no distinct polysaccharide peak or significant yield was obtained from the 0.3 mol/L NaCl elution step, thus resulting in the separation into three fractions (Figure 3A): MSP-1 (eluted with water), MSP-2 (eluted with 0.1 M NaCl), and MSP-3 (eluted with 0.2 M NaCl). Their yields were 33.2%, 12.2%, 10.3% (based on the dry weight of MSP). The highest-yielding MSP-1 was gathered for further purification with a Sephacryl S-400HR column. Figure 3B exhibited that a major elution peak was obtained, namely, MSP-1-1, with a yield of 51.6% (relative to the dry weight of MSP-1) and a purity of 94.8%. In this study, we focused on MSP-1-1.

#### 3.1.2. Spectral Analysis of MSP-1-1

**Figure 4:**
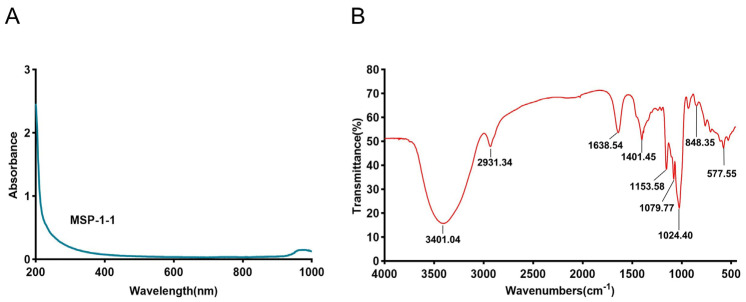
(**A**) UV-vis spectrum of MSP-1-1; (**B**) FT-IR spectrum of MSP-1-1.

The UV–vis spectra of MSP-1-1 exhibited no absorption peaks at 260 and 280 nm (Figure 4A), suggesting that neither nucleic acids nor proteins were present in the sample. The FT- IR spectrum of MSP-1-1 is presented in Figure 4B. At 3401.04 cm^−1^, the strong absorption band was ascribed to O–H stretching vibration, while the faint absorption band at 2931.34 cm^−1^ was associated with the C–H stretching vibration. The highest intensity absorption band at 1638.54 cm^−1^ was induced by the stretching motion of the C=O bond [17]. At 1153.58, 1079.77, and 1024.40 cm^−1^, the vibrations indicate the bending vibrational patterns of C-O bonds extending within the pyranose ring. Moreover, the highest intensity absorption band at 848.35 cm^−1^ suggests the existence of α-glycosidic bonds [26], which were confirmed via NMR spectral analysis.

#### 3.1.3. Mw and Monosaccharide Composition of MSP-1-1

**Figure 5:**
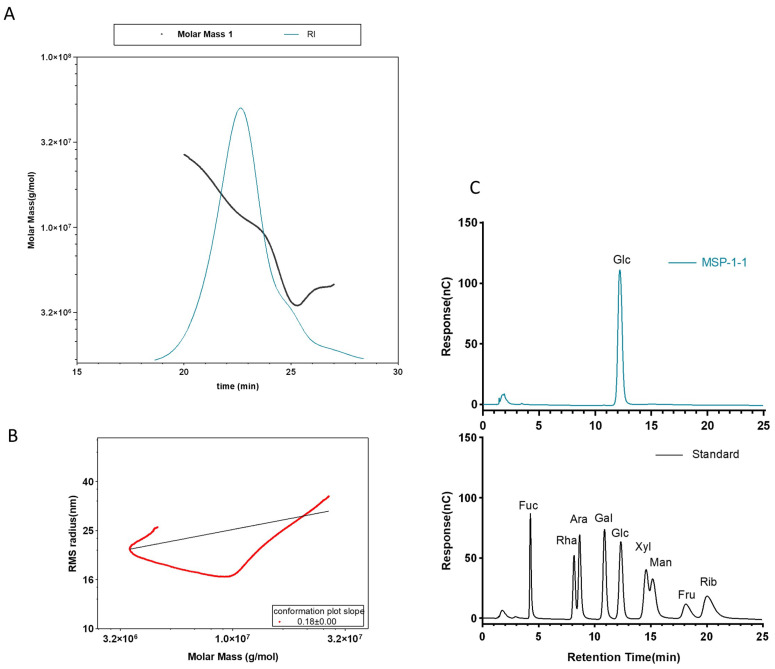
(**A**) Mw distribution of MSP-1-1; (**B**) molecular conformation of MSP-1-1; (**C**) ion chromatogram of standard monosaccharides and MSP-1-1.

The Mw of MSP-1-1 was 1.2 × 10^7^ Da, with a polydispersity index (Mw/Mn) of 1.25, close to 1, indicating good homogeneity of MSP-1-1, and similarly, the analyzed data from the RI detector in Figure 5A demonstrated the homogeneity of MSP-1-1 with a single symmetrical peak. As illustrated in Figure 5B, the plot of log (R.M.S. Radius, nm) against log (Molar Mass, g/mol) yielded a slope of 0.18. The slope of this double-logarithmic plot reflects the chain conformation of macromolecules: a slope of ~0.33 is characteristic of a random coil structure in a good solvent, while a slope < 0.33 corresponds to a curled and compact spherical conformation. The slope of 0.18 (smaller than 0.33) thus confirms that MSP-1-1 adopts a curled and compact spherical structure in aqueous solution [27]. Note that the minor phenomenon of one molar mass corresponding to two RMS radii at the lower molar mass range is common in polysaccharide MALLS analysis, attributed to conformational heterogeneity without affecting the overall trend [28]. Ion chromatography analysis in Figure 5C showed a single symmetric peak at 12.192 min, confirming the high monosaccharide homogeneity of MSP-1-1.

#### 3.1.4. Methylation Analysis of MSP-1-1

Sugar residues in MSP-1-1 were qualitatively and quantitatively detected via methylation reaction, and the results are shown in Table 1. We found that MSP-1-1 contained a total of four linkages, which included 4-Glc(*p*) (75.92%), t-Glc(*p*) (13.89%), 4,6-Glc(*p*) (8.57%) and a small amount of 3,4-Glc(*p*) (1.62%). This result was consistent with the cross-validated monosaccharide component analysis, which together confirmed that MSP-1-1 is a glucan. Critically, this analysis provided a quantitative profile of glycosidic linkage types and their molar ratios, offering the essential architectural blueprint of the polysaccharide’s structure.

#### 3.1.5. NMR Spectrum Analysis of MSP-1-1

**Figure 6:**
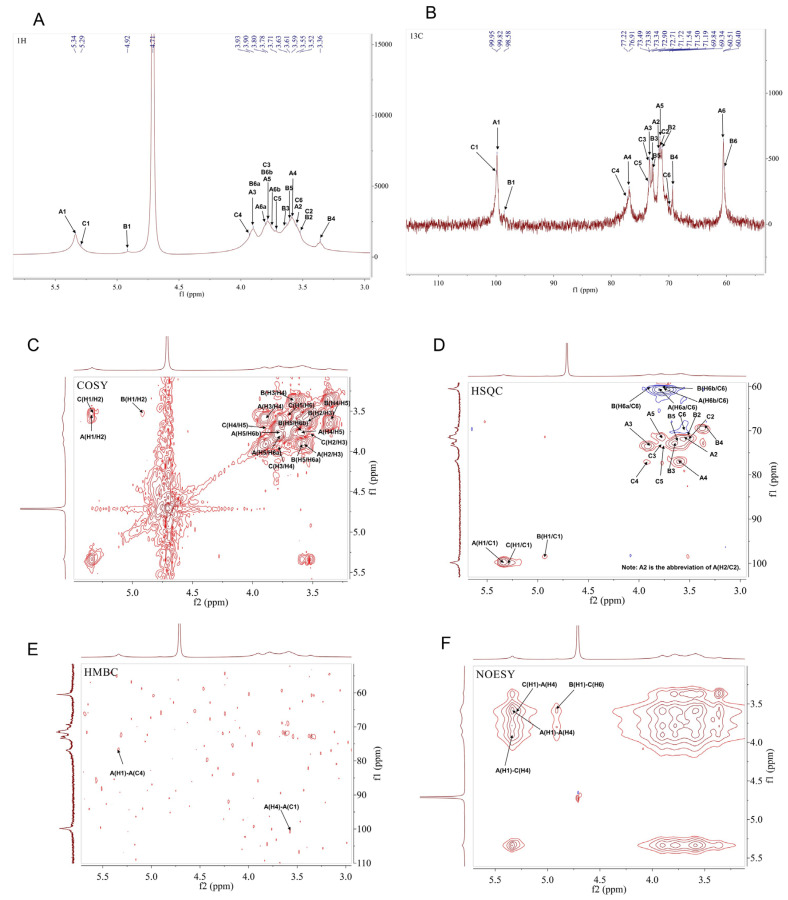
NMR spectra of MSP-1-1. (**A**) 1H-NMR; (**B**) 13C-NMR; (**C**) COSY; (**D**) HSQC; (**E**) HMBC; (**F**) NOESY.

Figure 6A,B show the 1D NMR (^1^H NMR and ^13^C NMR) spectra of MSP-1-1. Three distinct anomeric proton/carbon signals were observed in MSP-1-1, corresponding to δ 5.34/99.82 ppm, δ 4.92/98.58 ppm, and δ 5.29/99.95 ppm, which were, respectively, assigned to the H1/C1 signals of residues A, B, and C. Additionally, the anomeric proton signals of MSP-1-1 were primarily distributed within the 4.3–5.4 ppm range, and the configurations of these residues were determined to be *α*-type.

The chemical shifts of the three residues in MSP-1-1 were further characterized by combining 2D NMR spectra (COSY and HSQC). Six cross-peaks corresponding to residue A were observed in the COSY spectrum (Figure 6C), δ 5.34/3.56 ppm, δ 3.56/3.9 ppm, δ 3.9/3.59 ppm, δ 3.59/3.77 ppm, δ 3.77/3.8 ppm, and δ 3.77/3.74 ppm, which were consistent with the H2-H6a/b shifts of residue A listed in Table 2. In the HSQC spectrum (Figure 6D), seven cross-peaks were detected for residue A: δ 5.34/99.82 ppm, δ 3.56/71.72 ppm, δ 3.9/73.34 ppm, δ 3.59/76.91 ppm, δ 3.77/71.54 ppm, δ 3.80/60.51 ppm, δ 3.74/60.51 ppm. These peaks correspond to H1/C1, H2/C2, H3/C3, H4/C4, H5/C5, H6a/C6, and H6b/C6, respectively. Notably, the chemical shifts of C1 and C4 exhibited downfield shifts, which suggests that substitution occurred at the O-1 and O-4 positions of the sugar ring. When paired with methylation analysis outcomes and prior literature reports [24], we deduced that residue A likely corresponds to →4)-α-D-Glcp-(1→. The chemical shift data for residues B and C in MSP-1-1 are summarized in Table 2.

**Figure 7:**
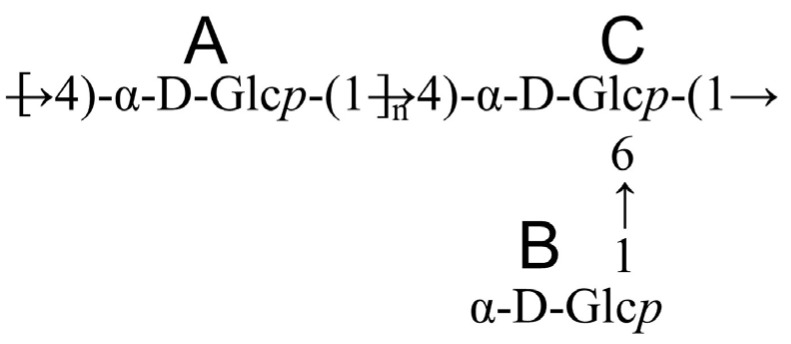
Proposed structural model of MSP-1-1. Notes: A and C represent the backbone α-D-Glc*p* residues; B represents the branched α-D-Glc*p* residue attached to the O-6 position of the backbone residue.

Finally, HMBC and NOESY spectra were combined to analyze the spatial cross-signals between distinct sugar residues, thereby identifying their glycosidic linkage configurations. The HMBC spectrum (Figure 6E) revealed the following cross-peak signals: A-H1/A-C4 (δ 5.34/76.91 ppm) and A-C1/A-H4 (δ 99.82/3.59 ppm). The NOESY spectrum (Figure 6F) revealed additional through-space correlations: A-H1/A-H4 (δ 5.34/3.59 ppm), A-H1/C-H4 (δ 5.34/3.93 ppm), B-H1/C-H6 (δ 4.92/3.56 ppm), and C-H1/A-H4 (δ 5.29/3.59 ppm). In summary, NMR spectroscopy elucidated the anomeric configuration (α), confirmed the specific linkage positions, and, most importantly, established the sequence and three-dimensional spatial relationships between residues, which are crucial for assembling the connectivity pattern. Integrated analysis of 1D/2D NMR and methylation data supported a structural model for MSP-1-1, featuring a backbone of →4)-α-D-Glc*p*-(1→ linkages with occasional branching via →4,6)-α-D-Glc*p*-(1→ residues. The branches were identified as single α-D-Glc*p*-(1→ units attached to the O-6 position of these branching residues. This proposed structure is summarized in Figure 7.

#### 3.1.6. Micromorphology Structure of MSP-1-1

**Figure 8:**
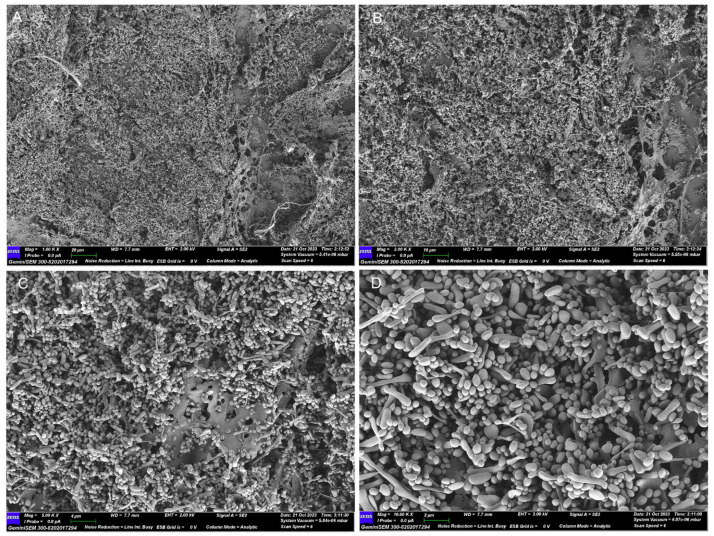
SEM images of MSP-1-1at (**A**) (1000×); (**B**) (2000×); (**C**) (5000×); (**D**) (10,000×).

SEM images at different magnifications (Figure 8) revealed that MSP-1-1 exhibited short rod-like and spherical morphologies, with particles clustered in aggregates.

### 3.2. Immunomodulatory Activity in CTX-Treated Mice

#### 3.2.1. Effect of MSP-1-1 on Immune Organs

**Figure 9:**
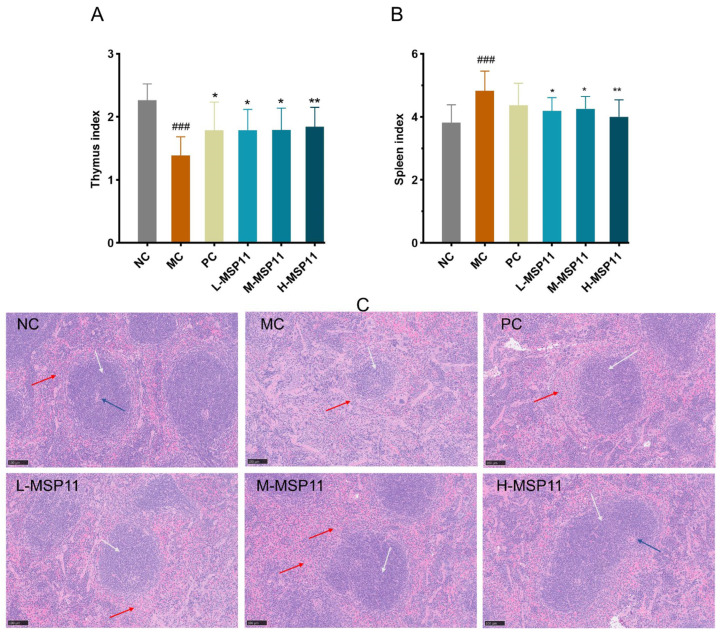
Effects of MSP-1-1on immune organ indices. (**A**) Thymus index (n = 12, mean ± SD); (**B**) spleen index (n = 12, mean ± SD); (**C**) HE staining of spleen section (original magnification: ×20, scale bar = 100). Statistical significance: ### *p* <  0.001 (vs. NC group); * *p* <  0.05, ** *p* <  0.01 (vs. MC group).

As illustrated in Figure 9A, the thymus index of the MC group was notably lower than that of the NC group—this demonstrates that CTX induced thymus atrophy in mice and successfully established the immunosuppression model. Following the intervention of MSP-1-1, subsequently, the thymus index increased significantly, suggesting that MSP-1-1 alleviates CTX-induced thymus atrophy and may regulate central immunity. As shown in Figure 9B, the mice in the MC group showed a significantly elevated spleen index, indicating that CTX-induced immune suppression led to compensatory splenomegaly, consistent with prior research by Huang et al. [29]. However, MSP-1-1 treatment alleviated splenic enlargement in immunosuppressed mice, thereby resulting in a significantly reduced splenic index.

As shown in Figure 9C, the boundary between the white pulp (indicated by white arrows) and red pulp (marked by red arrows) in the spleens of mice from the NC group was clearly defined. The intense staining of the white pulp signaled a high density of lymphocytes, with well-organized lymphoid follicles and clearly visible central arteries (indicated by blue arrows), demonstrating a normal splenic architecture. In contrast, the MC group exhibited an indistinct boundary between white and red pulp, accompanied by a significant reduction in lymphocyte density and abnormal lymphocyte distribution. Additionally, the lymphoid follicles displayed irregular morphology, suggesting cyclophosphamide-induced structural disruption. Compared with the MC group, both the PC group and the three MSP-1-1-treated groups showed more distinct separation between white and red pulps, along with enhanced staining intensity of the white pulp, showing varying degrees of structural recovery. Notably, MSP-1-1 treatment significantly attenuated cyclophosphamide-induced splenic structural alterations in a dose-dependent manner, with higher doses demonstrating near-complete restoration of splenic architecture, closely resembling the NC group.

#### 3.2.2. Effect of MSP-1-1 on Hematological Indices

**Figure 10:**
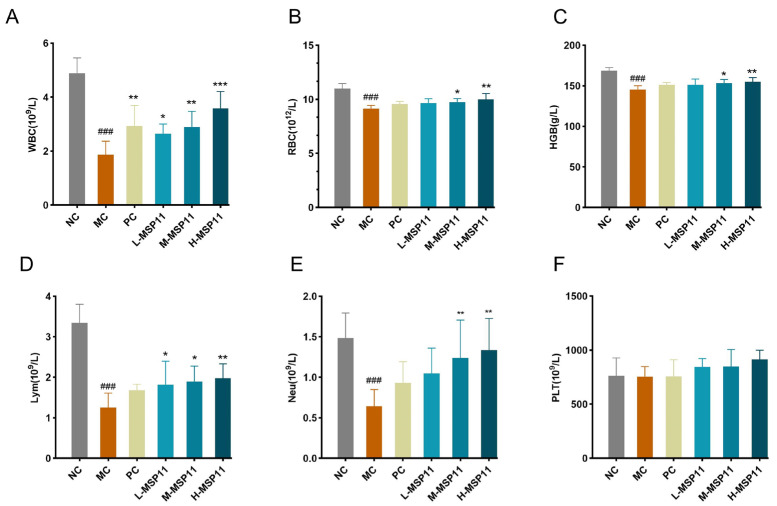
Effects of MSP-1-1 on hematological indices (n = 5, mean ± SD). (**A**) WBC; (**B**) RBC; (**C**) HGB; (**D**) Lym; (**E**) Neu; (**F**) PLT. Statistical significance: ### *p* <  0.001 (vs. NC group); * *p* <  0.05, ** *p* <  0.01, *** *p* <  0.001 (vs. MC group).

The hematopoietic activity of glucan was typically evaluated by analyzing peripheral blood hematological parameters. As shown in Figure 10A–F, CTX treatment significantly decreased WBC, RBC, HGB, Lym, and Neu levels. In contrast, medium and high doses of MSP-1-1 significantly increased these hematological parameters. However, no statistically meaningful variations in PLT were detected across the different experimental groups.

#### 3.2.3. Effect of MSP-1-1 on Monocyte–Macrophage Function and Splenic Lymphocyte Proliferation

**Figure 11:**
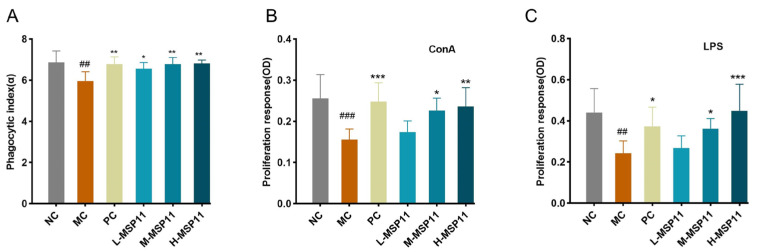
Effects of MSP-1-1 on macrophage carbon clearance capacity and ConA/LPS-induced splenocyte proliferation in CTX-induced immunosuppressed mice. (**A**) Carbon clearance capacity (n = 4, mean ± SD); (**B**) ConA -induced T lymphocyte proliferation in splenocytes (n = 5, mean ± SD); (**C**) LPS-induced B lymphocyte proliferation in splenocytes (n = 5, mean ± SD). Statistical significance: ## *p* <  0.01, ### *p* <  0.001 (vs. NC group); * *p* <  0.05, ** *p*  <  0.01, *** *p* <  0.001 (vs. MC group).

Macrophage carbon clearance capacity and phagocytic activity are established indicators of nonspecific immune function. As shown in Figure 11A, CTX treatment significantly decreased the carbon particle clearance rate in mice, which was reversed by MSP-1-1 intervention. ConA and LPS are mitogens that selectively stimulate T and B lymphocyte proliferation, respectively [30]. CTX significantly inhibited T and B lymphocyte proliferation, while this suppressive effect was weakened by medium and high doses of MSP-1-1 (Figure 11B,C). In conclusion, these findings indicate that MSP-1-1 alleviates CTX-induced immunosuppression through enhanced macrophage phagocytosis and restored T and B lymphocyte proliferation.

#### 3.2.4. Effect of MSP-1-1 on Splenic T Lymphocyte Subpopulations

**Figure 12:**
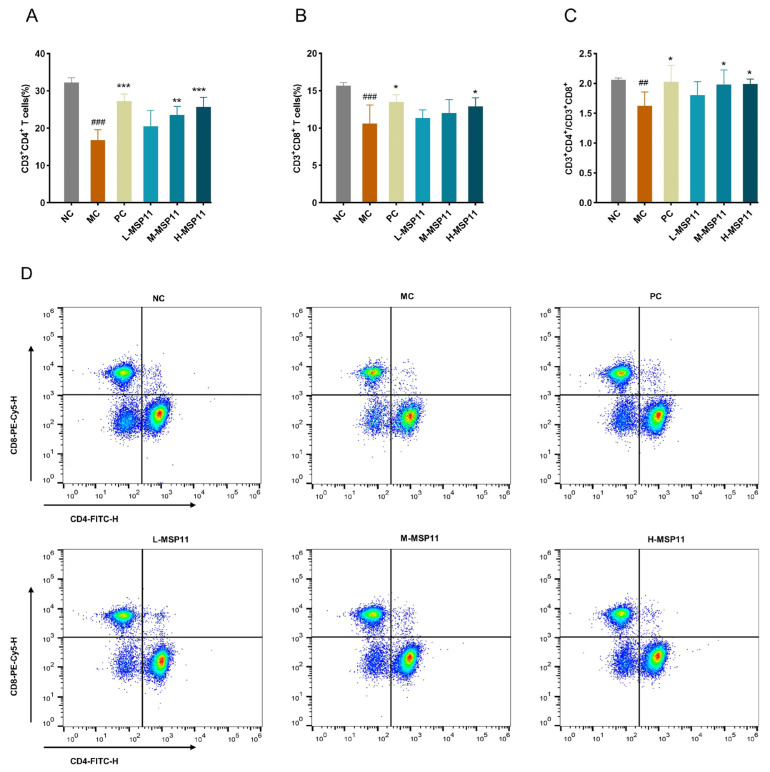
Effects of MSP-1-1 on the proportion of splenic CD4 or CD8T cells. (**A**) Proportion of CD3^+^CD4^+^ T cells (n = 5, mean ± SD); (**B**) proportion of CD3^+^CD8^+^T cells (n = 5, mean ± SD); (**C**) the ratio of CD3^+^CD4^+/^CD3^+^CD8^+^T cells (n = 5, mean ± SD). (**D**) Flow cytometry. Statistical significance: ## *p* <  0.01, ### *p* <  0.001 (vs. NC group); * *p* <  0.05, ** *p* <  0.01, *** *p* <  0.001 (vs. MC group).

Flow cytometry was applied to detect splenic lymphocyte subpopulations in immunosuppressed mice, and the findings are illustrated in Figure 12. CTX significantly diminished the proportion of CD3^+^CD4^+^ T cells, CD3^+^CD8^+^ T cells, and the CD3^+^CD4^+^/CD3^+^CD8^+^ ratio. However, after MSP-1-1 intervention, both the count of CD3^+^CD4^+^ T cells and the CD3^+^CD4^+^/CD3^+^CD8^+^ ratio were markedly elevated in the M-MSP11 and H-MSP11 groups; meanwhile, the proportion of CD3^+^CD8^+^ T cells was significantly increased in the H-MSP11 group. This suggests that high doses of MSP-1-1 regulate immunity by balancing T lymphocyte subpopulations in mice.

#### 3.2.5. Effect of MSP-1-1 on Serum and Thymus Immunoglobulin Levels

**Figure 13:**
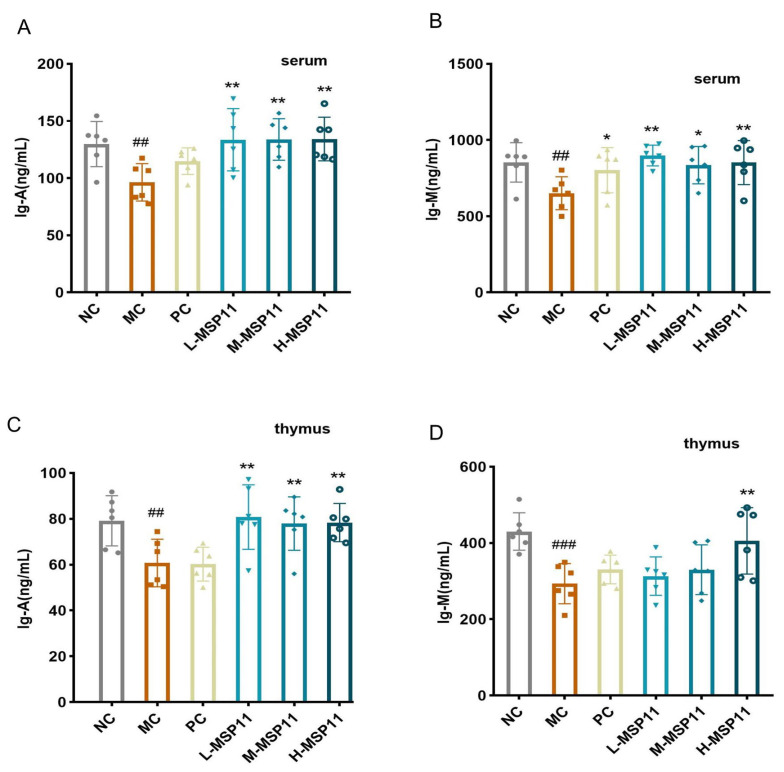
Effects of MSP-1-1 on immunoglobulin levels in serum and thymus (n = 6, mean ± SD). (**A**) Serum IgA concentration; (**B**) serum IgM concentration; (**C**) thymus IgA concentration; (**D**) thymus IgM concentration. Statistical significance: ## *p*  <  0.01, ### *p*  <  0.001 (vs. NC group); * *p*  <  0.05, ** *p*  <  0.01 (vs. MC group).

ELISA was used to measure serum and thymus immunoglobulin (IgA and IgM) levels. The results showed that cyclophosphamide significantly reduced immunoglobulin levels in the serum of the MC group, whereas MSP-1-1 administration significantly counteracted this reduction. After 24 days of oral MSP-1-1 treatment, all tested doses significantly increased serum IgA and IgM levels (Figure 13A,B). A similar trend of immunoglobulin changes was found in the thymus. As illustrated in Figure 13C,D, CTX caused a significant decrease in thymic IgA and IgM levels. However, intervention with three doses of MSP-1-1 elevated IgA levels, while only the high-dose MSP-1-1 group showed a significant increase in IgM levels.

## 4. Discussion

In recent years, edible fungus glucans, as natural compounds, have been extensively researched as supplements by scholars due to their safety and remarkable biological activities [2]. In this research, we found that MSP-1-1 significantly enhanced immune function in CTX-induced immunosuppressed mice after 24 days of continuous administration.

Firstly, we obtained homogeneous glucan fractions from *M. sextelata* fruiting bodies and characterized their structural properties. Molecular weight is a critical parameter of glucans; it corresponds to the length of the molecular chain and is closely linked to the biological activities of these compounds [31,32]. Previous studies have reported the isolation of immunologically active glucans from edible fungi, with molecular weights ranging from 10^4^ to 10^7^Da. The molecular weight of MSP-1-1 in our study is consistent with these previous findings [33].

The physicochemical properties and physiological functions of glucans are affected by their separation and purification methods [18,34]. Structural analysis revealed that MSP-1-1 was composed entirely of glucose. A proposed model features a main chain of →4)-α-D-Glc*p*-(1→ linkages and occasional branches at →4,6)-α-D-Glc*p*-(1→ residues, with α-D-Glc*p*-(1→ side chains attached at their O-6 position. The monosaccharide composition of MSP-1-1 aligns with several reported Morchella glucans [6], which also exhibit a (1→4)-linked backbone. This consistency suggests a conserved structural motif within the genus that may be associated with certain bioactivities, such as immune modulation through interactions with PRRs [33]. However, discrepancies exist in the literature regarding the monosaccharide composition of *Morchella* polysaccharides: Peng et al. isolated a polysaccharide from *Morchella importuna* (a different species in the same genus as *M. sextelata*) consisting of glucosamine, galactose, glucose, and mannose [7], while another study reported a *Morchella esculenta* polysaccharide extracted via alkali solution, which contained mannose, glucose, and galactose [35]. These variations suggest that monosaccharide composition may depend on multiple factors, including fungal species, extraction protocols, geographical origin, and processing conditions.

Immune organs play essential roles in immunomodulation, and their immunomodulatory activity is strongly correlated with the changes in immune organ indices [36]. Cyclophosphamide affects the spleen through two distinct mechanisms. First, as an immune organ, the spleen undergoes atrophy due to cyclophosphamide’s inhibitory effects, resulting in decreased organ weight and index. Second, as a secondary hematopoietic organ, the spleen activates extramedullary hematopoiesis when bone marrow function is suppressed, leading to compensatory proliferation and increased organ weight/index [37]. The net effect on spleen morphology depends on the balance between these opposing processes, which varies with dosage and treatment duration. Our study demonstrated that MSP-1-1 effectively mitigated CTX-induced splenomegaly and thymic atrophy in mice, consistent with previous findings regarding Mesona chinensis Benth polysaccharides in CTX-induced immunosuppression [29]. The bone marrow, as the primary site of hematopoiesis and a central immune organ, plays crucial roles in immunity and defense [38]. A well-documented side effect of cyclophosphamide chemotherapy is bone marrow suppression [39]. Notably, our results revealed that MSP-1-1 significantly ameliorated cyclophosphamide-induced bone marrow suppression.

Immune cells are central to the body’s immune defense, encompassing immune cell subsets like lymphocytes (e.g., T and B cells), dendritic cells (DCs), mononuclear/macrophages, and natural killer cells (NKs) [40]. MSP-1-1 significantly improved the carbon clearance capacity of macrophages in CTX-induced immunosuppressed mice. T and B lymphocytes act as the primary immune cells of the body, mediating adaptive and humoral immune responses, respectively [41]. The proliferation of T and B lymphocytes stimulated by ConA and LPS served as the basis for evaluating lymphocyte immune activity [42]. In this research, CTX inhibited the proliferative capacity of lymphocytes in response to ConA and LPS stimulation, but the proliferation rate of splenic lymphocytes was dramatically increased after MSP-1-1 intervention. This finding was consistent with that of a study on Mesona chinensis Benth polysaccharide [29].

CD3 is the main surface marker of T cells, reflecting the immune functional status of the body’s cells [43,44]. CD4 and CD8 are surface molecules of T cells, participating in adaptive immune responses. CD4^+^ T lymphocytes represent helper cells that secrete large amounts of cytokines, helping to maintain immune self-tolerance [45,46]. CD8^+^ T lymphocytes function as effector cells [47]. Notably, the CD4^+^/CD8^+^ ratio is considered a more sensitive indicator of cell-mediated immune responses than individual subset analysis. Our research found that the proportions of CD3^+^CD4^+^ cells, CD3^+^CD8^+^ cells, and the CD3^+^CD4^+^/CD3^+^CD8^+^ were significantly reduced after intraperitoneal injection of cyclophosphamide, but this trend was reversed after MSP-1-1 intervention. Previous studies have shown that CTX reduces the CD3^+^CD4^+^/CD3^+^CD8^+^ ratio in mice, while oral administration of polysaccharides and ginsenosides can counteract this effect [48]. Moreover, complex probiotics have demonstrated efficacy in restoring cyclophosphamide-induced reductions in CD4^+^ and CD8^+^ T cell proportions [49]. As a critical mediator of humoral immunity, immunoglobulin provides protective functions by neutralizing foreign pathogens and activating complement [50]. Our results revealed that MSP-1-1 significantly reversed the CTX-induced decline in IgA and IgM levels. In summary, MSP-1-1 effectively ameliorated CTX-induced cellular immune dysfunction by restoring lymphocyte subset homeostasis and augmenting humoral immunity via enhanced production of specific immunoglobulins.

## 5. Conclusions

In summary, MSP-1-1 is a glucan isolated from *M. sextelata*, with an Mw of 1.2 × 10^7^ Da. Structural analysis led to a proposed model in which the main chain is predominantly composed of →4)-α-D-Glc*p*-(1→ linkages, with occasional →4,6)-α-D-Glc*p*-(1→ residues serving as branch points. The branches are single α-D-Glc*p*-(1→ units linked at the O-6 position of these residues. MSP-1-1 could restore CTX-induced abnormalities in immune organ indexes and peripheral blood levels in vivo. In addition, MSP-1-1 significantly enhanced the phagocytic ability of macrophages and promoted the proliferation of splenic lymphocytes, while reversing the proportion of splenic lymphocyte subsets. It also elevated the concentrations of serum and thymic immunoglobulins (including IgA and IgM), thereby exerting immunomodulatory effects. In conclusion, this study demonstrates that MSP-1-1 is a natural compound with immunomodulatory activity, holding potential as an immunomodulatory agent for applications in health foods and pharmaceuticals. However, further validation in other animal models and more in-depth investigations into the underlying immunological mechanisms are still warranted.

## Figures and Tables

**Table 1:** Linkage-type analysis of MSP-1-1 conducted using GC–MS.

Linkage Pattern	Methylated Sugar	Mass Fragments (*m*/*z*)	Molar Ratio (%)	Retention Time (min)
t-Glc(*p*)	1,5-di-O-acetyl-2,3,4,6-tetra-O-methyl glucitol	87, 102, 118, 129, 145, 161, 162, 205	13.89	9.4
4-Glc(*p*)	1,4,5-tri-O-acetyl-2,3,6-tri-O-methyl glucitol	87, 102, 113, 118, 129, 162, 233	75.92	14.8
3,4-Glc(*p*)	1,3,4,5-tetra-O-acetyl-2,6-di-O-methyl glucitol	87, 118, 129, 143, 185, 203, 305	1.62	16.9
4,6-Glc(*p*)	1,4,5,6-tetra-O-acetyl-2,3-di-O-methyl glucitol	85, 102, 118, 127, 159, 162, 201, 261	8.57	19.0

**Table 2:** Assignment of ^1^H-NMR and ^13^C-NMR chemical shifts of MSP-1-1.

Code	Sugar Residues	Chemical Shifts (ppm)					
		H1/C1	H2/C2	H3/C3	H4/C4	H5/C5	H6a, b/C6
A	→4)-α-D-Glc*p*-(1→	5.34/99.82	3.56/71.72	3.9/73.34	3.59/76.91	3.77/76.91	3.80,3.74/60.51
B	α-D-Glc*p*-(1→	4.92/98.58	3.52/71.19	3.65/72.9	3.36/69.34	3.61/72.71	3.9,3.77/60.4
C	→4,6)-α-D-Glc*p*-(1→	5.29/99.95	3.51/71.5	3.92/73.38	3.77/77.22	3.71/72.78	3.56/69.84

## Data Availability

The original contributions presented in the study are included in the article; further inquiries can be directed to the corresponding author.
